# Metabolic Reprogramming in Adipose Tissue During Cancer Cachexia

**DOI:** 10.3389/fonc.2022.848394

**Published:** 2022-05-12

**Authors:** Bahar Zehra Camurdanoglu Weber, Dilsad H. Arabaci, Serkan Kir

**Affiliations:** Department of Molecular Biology and Genetics, Koc University, Istanbul, Turkey

**Keywords:** cancer cachexia, adipose tissue, lipolysis, lipogenesis, non-shivering thermogenesis, adipose tissue browning, adipokines, inflammation

## Abstract

Cancer cachexia is a disorder of energy balance characterized by the wasting of adipose tissue and skeletal muscle resulting in severe weight loss with profound influence on morbidity and mortality. Treatment options for cancer cachexia are still limited. This multifactorial syndrome is associated with changes in several metabolic pathways in adipose tissue which is affected early in the course of cachexia. Adipose depots are involved in energy storage and consumption as well as endocrine functions. In this mini review, we discuss the metabolic reprogramming in all three types of adipose tissues – white, brown, and beige – under the influence of the tumor macro-environment. Alterations in adipose tissue lipolysis, lipogenesis, inflammation and adaptive thermogenesis of beige/brown adipocytes are highlighted. Energy-wasting circuits in adipose tissue impacts whole-body metabolism and particularly skeletal muscle. Targeting of key molecular players involved in the metabolic reprogramming may aid in the development of new treatment strategies for cancer cachexia.

## Introduction

Cachexia is a complex disorder characterized by the loss of adipose and skeletal muscle tissues leading to chronic and involuntary weight loss that cannot be fully reversed by conventional nutritional support (1). Cachexia occurs in association with cancer and multiple other chronic diseases, including heart failure and kidney disease (2). The incidence of cachexia is particularly very high in cancer patients. About half of all cancer patients suffer from cachexia which is the direct cause of at least 20% of all cancer deaths (1, 3). Patients with pancreatic or gastric cancer have the highest frequency of weight loss at over 60-80% while the incidence of weight loss in patients with lung, colorectal or prostate cancer is above 50% (1, 3). Cachexia remains as a significant risk factor for survival as it reduces quality of life and leads to a poor response to therapies.

Cachexia ameliorates with the shrinkage of tumors and the treatment of underlying cancer to reverse cachexia is the best-thought management strategy so far. However, this method remains unsuccessful with advanced cancer types. Few anti-inflammatory drugs, mainly targeting tumor necrotic factor α (TNF-α) and interleukin-6 (IL-6), were tested clinically and the trials yielded unsatisfactory results. Most recent therapeutic approaches target decreased appetite in cancer patients to counteract weight loss. However, treatment of malnutrition or anorexia by increasing nutritional intake has not been able to completely reverse the wasting (4). Cancer cachexia is still very common, treatment options are inadequate and clinical markers are missing. Understanding the molecular and physiologic pathways involved in the etiology of cancer cachexia is urgently needed. At the present, cachexia is agreed to be an energy disorder, in which the peripheral energy metabolism plays a central role in elevated energy expenditure and excess catabolism with severe disruption of protein, lipid, and carbohydrate metabolism accompanied with chronic inflammation (1). Cancer cachexia affects multiple organs with most dramatic impacts on adipose and skeletal muscle tissues (5). In this review, we discuss how metabolic activities of different types of adipose tissue are altered by tumors and how this metabolic reprogramming contributes to the wasting problem.

## Adipose Tissue

Adipose tissue is a highly dynamic, metabolically active organ involved in a multitude of physiological processes. Adipose tissue contains many cell types including endothelial cells, fibroblasts, macrophages and other immune cells along with adipocytes (6). Adipose tissue has been classically considered as a fat reservoir for energy storage and thermal insulation (7). Since the identification of leptin (8), adipose tissue has been established as an endocrine organ that expresses and secretes a variety of proteins named ‘adipokines’ allowing it to communicate with other organs, such as muscle, pancreas, liver and brain. Besides, certain adipose depots function as an energy-consuming tissues *via* carrying out non-shivering thermogenesis that promotes weight loss (9–11).

Three distinct types of adipose tissue have been characterized according to their morphology and function; classical white adipose tissue (WAT) stores lipids and secretes endocrine factors, classical brown adipose tissue (BAT) executes non-shivering thermogenesis, and beige/brite adipose tissue (BeAT) participates in adaptive thermogenesis. Three main types of adipocytes reside within these tissues: i) White adipocytes with a unilocular lipid droplet are present in WAT and BeAT tissues, these cells function in energy deposition by storing triglycerides (TG) when energy input exceeds expenditure (lipogenesis) and in energy mobilization by releasing fatty acids (FA) and glycerol into the circulation *via* triglyceride hydrolysis (lipolysis) (6, 7). WAT is primarily located at subcutaneous, abdominal, inguinal, and gonadal regions (6, 12). ii) Brown adipocytes consist of multilocular lipid droplets and a high number of mitochondria expressing uncoupling protein-1 (UCP-1) which uncouples oxidative phosphorylation from adenosine triphosphate (ATP) synthesis by allowing leakage of protons through the inner membrane. This process mediates non-shivering thermogenesis and generates heat instead of ATP. Consequently, BAT is mainly implicated in thermogenesis and energy balance together with lipid oxidation (6, 7). Cold exposure and feeding promote release of norepinephrine from the sympathetic nervous system leading to activation and expansion of BAT (11). Catecholamines released by sympathetic nerve terminals act on β-adrenergic receptors to stimulate BAT thermogenesis. Classical BAT is typically located in the interscapular region and is abundantly detected in human infants and small rodents (11). iii) Beige adipocytes were identified more recently as clusters of UCP1-expressing cells located in WAT. Beige cells show similar morphology to brown adipocytes with multilocular lipid droplets and high mitochondrial content. Although, beige cells express thermogenesis-related genes at low levels basally, their expression (primarily UCP-1) can be potently induced by cold exposure, exercise, agonists of β-adrenergic receptors or peroxisome proliferator-activated receptor-γ (PPARγ). This process, also termed as ‘browning’ of WAT, results in activation of cellular respiration and energy expenditure by adaptive thermogenesis (11, 13). In adult humans, thermogenic beige/brown adipocytes are distributed throughout the cervical, supraclavicular, axillary, paravertebral, mediastinal, and upper abdominal regions (6, 14).

Several alterations have been suggested as the cause of reduction in adipose tissue mass during cancer cachexia, including, increased lipolysis in adipocytes, decreased lipogenesis, abnormal inflammatory response, impaired adipocyte formation and enhanced thermogenic activity (15–24). All of these changes are linked to cachexia-driven metabolic reprogramming in adipose tissue and are featured in this review.

## Lipolysis and Lipogenesis

Increased lipid utilization from adipose depots was indicated in tumor-bearing cachectic mice leading to depletion of lipid stores and loss of fat mass (16). Enhanced lipolysis has been suggested as one of the characteristics of cachexia in both cancer patients and rodent models of cachexia (16, 18, 25). Lipolysis is a process orchestrated by three lipases sequentially; adipocyte triglyceride lipase (ATGL), hormone sensitive lipase (HSL), and monoacylglycerol lipase (MGL) (26). Increased expression levels of HSL mRNA and protein is detected in WAT of cachectic cancer patients, while body fat was reduced and lipolytic activity was increased (18). Cancer patients also exhibited a correlation between the serum FA levels and expression of *HSL* mRNA in the adipose tissue (27). Additionally, increased protein levels and phosphorylation of HSL which drives lipolytic activity were detected in murine cachexia tumor models (19, 21). Upregulation of ATGL protein was also detected in WAT of cachectic cancer patients (28). Genetic deletion of ATGL or HSL in tumor-bearing mice preserved WAT and muscle mass by suppressing lipolysis in WAT and proteasomal protein degradation in skeletal muscle (16) (**Table 1**). The mechanisms and factors governing altered HSL and ATGL activity are still needed to be elucidated (16). Additionally, Cell Death–Inducing DNA Fragmentation Factor-α-Like Effector A (CIDEA) located on the surface of lipid droplets was also associated with lipolysis. Its expression was found to be increased in WAT of cachectic cancer patients and correlated with elevated levels of FAs and weight loss (29). CIDEA was reported to be essential for tumor-induced lipolysis by interacting and destabilizing AMP kinase (AMPK) in adipose tissue (30) (**Table 1**). However, CIDEA was also implicated in the inhibition of lipolysis as its deficiency in rodents led to increased FA oxidation in BAT and acceleration of lipolysis (29). Further studies are needed to understand the nature of CIDEA involvement in lipolysis.

**Table 1:** Summary of catabolic factors regulating the metabolic reprogramming in adipose tissue.

Secreted factor	Source	Name	Metabolic role in adipose tissue
Yes	Tumor	PTHrP	Browning
			Lipolysis
	Tumor	GDF15	Lipolysis
			Browning
	Tumor	ZAG	Lipolysis
	Adipocyte		Browning
	Tumor	LIF	Lipolysis
	Tumor	Adrenomedullin	Lipolysis
	Adipocyte		
	Tumor	TNF-α	Lipolysis
	Immune cells		
			Inflammation
	Adipocyte		
	Tumor	IL-6	Lipolysis
	Immune cells		Browning
	Adipocyte		Inflammation
	Adipocyte	Leptin	Lipolysis
	Sympathetic neurons	Catecholamines	Browning
			Lipolysis
	Brain	Natriuretic peptides	Lipolysis
	Heart		
	Adrenal gland	Glucocorticoids	Lipolysis
	Pancreas	Insulin	Lipogenesis
No	Adipocyte	CIDEA	Lipolysis
		HSL	Lipolysis
		ATGL	Lipolysis
		LPL	Lipogenesis
		FAS	Lipogenesis
		TLR4 signaling	Lipolysis
			Browning
			Immune cell composition

**Figure 1:**
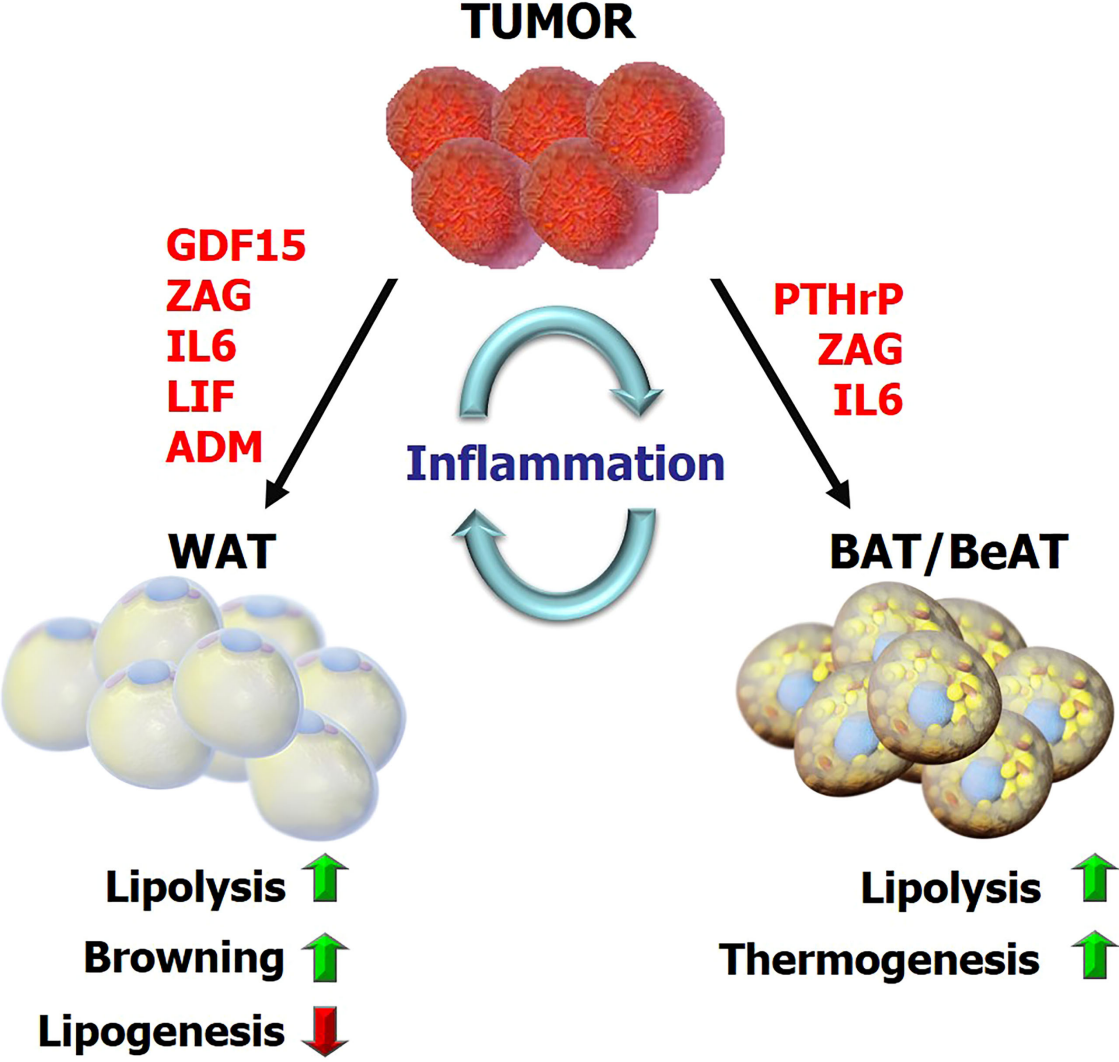
The overview of metabolic reprogramming in adipose tissues during cancer cachexia Tumor-derived factors and tumor-host interactions impact the metabolic programs in adipose tissue including lipolysis, lipogenesis, inflammation, browning and adaptive thermogenesis. This reprogramming subsequently promotes energy-wasting processes contributing to cancer cachexia. GDF15, growth differentiation factor 15; ZAG, Zinc-α2-glycoprotein; LIF, leukemia inhibitory factor; PTHrP, parathyroid-hormone-related protein; ADM, adrenomedullin.

The lipolytic activity can be induced during cancer cachexia by various signals including catecholamines, natriuretic peptides, glucocorticoids, adipokine Zinc-α2-glycoprotein (ZAG), TNF-α and IL-6 (18, 23, 31, 32). Upregulation of ZAG expression and secretion was reported in WAT of cachectic gastrointestinal and pancreatic cancer patients (33). A positive correlation was identified between ZAG expression in adipose tissue, increased fasting serum glycerol levels and weight loss in cachectic patients (33). ZAG stimulates lipolysis in adipocytes *via* activation of β3-adrenergic receptors (β3ARs) and cAMP pathway (34) (**Table 1**). Additionally, ZAG-overexpressing transgenic mice exhibited decreased body weight and WAT mass, elevated *HSL* mRNA levels in adipose tissue, and sensitization to catecholamines (33, 35) (**Figure 1**). Increased IL-6 levels and a positive correlation between serum IL-6 and free FAs was also reported in early and late-stage cachexia patients indicating a possible influence on WAT lipolysis (23). Moreover, treatment of cachectic mice with an anti-IL-6 receptor antibody inhibited lipolysis and rescued the loss of WAT (23) (**Figure 1**; **Table 1**). Pro-inflammatory cytokine TNF-α was also implicated in cancer cachexia and shown to promote lipolysis in rodents and human adipocytes (36, 37) (**Table 1**). However, its relevance in adipose atrophy is still ambiguous as serum TNF-α was unchanged in cachectic cancer patients and did not correlate with weight loss (20, 38, 39). Recently, leukemia inhibitory factor (LIF), a member of the IL-6 family of cytokines, was indicated in adipocyte lipolysis. LIF is secreted from tumors and promotes loss of fat mass and body weight (40, 41) (**Figure 1**; **Table 1**). Moreover, this effect can be counterbalanced by reduced leptin signaling in a murine colon adenocarcinoma model (40). These effects are likely mediated by JAK kinase as administration of JAK inhibitors suppressed LIF-associated adipose wasting and improved survival in cachectic mice (41). Recently, circulating growth differentiation factor 15 (GDF15) was found to be elevated in cachectic cancer patients (42). GDF15 levels correlated with cachexia and a poor survival. GDF15 administration in mice induces ATGL and HSL expression in WAT (42). ATGL-knockout mice do not show loss of body weight or fat mass upon overexpression of GDF15 indicating GDF15-driven wasting depends on ATGL (42). Treatment of tumor-bearing mice with a specific antibody against GDF15 receptor inhibited lipid mobilization and oxidation in adipose tissue, preserved muscle mass, and prevented weight loss (42) (**Table 1**). Thus, tumor-derived GDF15 induces lipolytic activity and likely contributes to cancer cachexia (42) (**Figure 1**).

While increased lipolysis contributes to adipose tissue loss, decreased lipogenesis was also implicated in cancer cachexia. Reduced levels of fatty acid synthase (FAS) and lipoprotein lipase (LPL were demonstrated in adipose tissue from cachectic cancer patients and animal tumor models (43–45) (**Figure 1**). In colorectal cancer patients, decreased activity of LPL and FAS was shown in the adipose tissue adjacent to the tumor (44). On the contrary, increased lipolysis and unaltered adipocyte lipogenesis was reported in cachectic gastrointestinal cancer patients with normal insulin-stimulated lipogenesis (20) (**Table 1**). Insulin is known to promote lipid synthesis and storage while prominently inhibiting lipolysis and FA oxidation (46) (**Table 1**). Abnormal insulin response, insulin resistance and reduced circulating insulin levels were detected in cachectic cancer patients, potentially causing a shift in metabolic balance resulting in elevated lipolysis (47). Alterations in adipose tissue gene expression profiles including genes involved in adipocyte differentiation and lipogenic enzymes were reported to contribute to malformations in adipocytes and cachexia-driven loss of adipose mass (17, 48). While further studies are needed to determine the role of lipogenesis in cancer cachexia, development of insulin resistance in cancer patients likely promotes a catabolic state inducing energy wasting.

## Adipose Tissue Thermogenesis and Browning

Activation of BAT has been implicated to promote hypermetabolism associated with the excessive weight loss of cancer patients (49, 50). Increased BAT thermogenic activity was also detected in various rodent models of cachexia (51–53). BAT activation was particularly reported in breast cancer patients where a robust association was seen in younger women (54). A direct correlation between BAT volume and mortality was described in a large group of cancer patients (55). However, two recent studies failed to demonstrate a link between BAT activation and weight loss (56, 57). Factors influencing detection of BAT activity using FDG-PET/CT scan were described in a large retrospective study (58). Further studies utilizing new technologies in BAT detection and considering various external influences are needed to validate the involvement of BAT thermogenesis in cancer cachexia (59).

Recent studies also indicated browning of WAT and the thermogenic activity of BeAT as important contributors to cachexia-associated weight loss (13, 15, 21, 60). Interestingly, WAT browning was detectable in pre-cachectic tumor-bearing mice before the loss of total body weight and skeletal muscle mass. It is associated with increased energy expenditure in tumor-bearing mice and increased expression of thermogenesis-related genes, including *Ucp-1* in WAT depots (13, 21). Elevated IL-6 was associated with activation of a browning program in WAT of cachectic mice while anti-IL-6 receptor antibody inhibited WAT browning in tumor-bearing mice (23) (**Figure 1**). Additionally, β3AR activation was linked to WAT browning while neutralization of IL-6 or inhibition of β3AR significantly ameliorated cancer cachexia (21) (**Table 1**). Furthermore, WAT biopsies collected from cachectic cancer patients demonstrated extensive UCP1 staining arguing a role for WAT browning (21). In fact, browning of WAT was reported in several mouse models of cachexia (15, 19, 21, 53). Particularly, elevated oxygen consumption as an indicator of hypermetabolism was detected in Lewis lung carcinoma (LLC) tumor-bearing mice which also exhibited WAT browning. Activation of a thermogenic program and upregulation of various genes involved in catabolism were reported in WAT of tumor-bearing mice (15). Interestingly, adipocyte-specific PRDM16 knockout mice in which thermogenic gene expression is impaired in BeAT were also found to be resistant to browning and tumor-induced weight loss (15, 61). Tumor-derived parathyroid-hormone-related protein (PTHrP) was elucidated to be responsible for WAT browning and wasting (15) (**Figure 1**; **Table 1**). Neutralization of PTHrP or deletion of its receptor (PTHR) in adipocytes prevented adipose tissue browning and wasting and also preserved muscle mass and strength in tumor-bearing mice (60). PTHrP expression was investigated in a cohort of patients diagnosed with metastatic non-small-cell lung cancer or colorectal cancer. Patients with high serum PTHrP concentrations were found to have significantly lower lean body mass and higher resting energy expenditure compared to patients lacking this protein in their blood (15). Additional studies analyzing large groups of cancer patients also reported a positive association between serum PTHrP and cancer-related weight loss, further supporting a role for PTHrP in wasting (62, 63). Additional studies are needed to uncover the therapeutic potential of targeting PTHrP in cachectic cancer patients.

Extracellular vesicles (EVs) are demonstrated to be critical for communication between tumor and adipose tissue in cancer cachexia. In fact, LLC cell-derived exosomes can induce lipolysis and browning. Inhibition of exosome generation/release can inhibit lipolysis and cachexia development in mice (64, 65). The inhibition of exosome release from tumor by a diuretic drug, amiloride, improved muscle and adipose wasting (66). PTHrP was detected in extracellular vesicles (EV) derived from tumors. These EVs carry the capacity to promote lipolysis and browning in adipose tissue, which can be reversed by knockdown or neutralization of PTHrP (67). Tumor-derived exosomes carrying miR-146b-5p also promotes adipose tissue loss and browning (68) and the circular RNA ciRS-133 down-regulated miR133 and induces browning process, through PRDM16 (69). Additionally, adrenomedullin containing exosomes derived from pancreatic cancer patients promoted lipolysis in murine and human adipocytes *via* activating MAPK and cAMP/PKA pathways. This effect was abrogated by inhibition of adrenomedullin receptor (70) (**Table 1**).

Other studies demonstrating enhanced WAT browning during cancer cachexia featured tumor-derived ZAG which triggers lipolysis and browning in a murine model *via* activating the β3AR pathway (71). Tumor-derived GDF15 was also shown to reduce fat mass by increasing expression of thermogenic genes in BAT and lipolytic genes in WAT and BAT, consistent with higher energy metabolism (72). Recently, increased neuronal catecholamine synthesis and secretion was linked to WAT browning and lipid catabolism *via* β-adrenergic activation of adipocytes in cachexia mouse models. Interleukin-4 receptor deficiency was shown to block alternative activation of macrophages, reduced sympathetic activity and WAT browning (19). Similarly, reduced catecholamine synthesis in peripheral dopamine β-hydroxylase (DBH)–deficient mice prevented cancer-induced WAT browning and adipose atrophy indicating the existence of an intraadipose macrophage-sympathetic neuron cross-talk (19). Mice lacking UCP-1 was used to study the role of adipose thermogenesis in cancer cachexia. Interestingly, these mice were found to be still prone to cachexia and adipose wasting. Therefore, additional pathways, such as AMPK signaling, may be involved in futile cycles capable of causing energy wasting in adipose tissue (30).

## Role of Adipokines in Cancer Cachexia

Cancer cachexia is associated with altered adipokine secretion. While the role of adipokine dysregulation in cachexia remains unclear, cachectic patients often show altered circulating levels of adipokines, including leptin, adiponectin and resistin (73). Leptin levels were found to be significantly decreased in cachectic cancer patients compared to both non-cachectic and healthy controls (74, 75). Animal studies have also shown that circulating leptin levels are decreased in the setting of tumor-induced cachexia (76). Administration of recombinant leptin to genetically obese mice reduces food intake, stimulates weight loss and energy expenditure (77). Additionally, leptin administration increases lipolysis and expression of UCP1 in adipose tissues (77, 78) (**Table 1**). How reduced leptin levels may potentially contribute to cachexia is not known. A negative correlation between resistin levels and body mass index (BMI) was reported (73). However, other studies failed to detect a difference in serum resistin levels in cachectic cancer patients (75, 79, 80).

Several clinical studies indicated a positive correlation between circulating adiponectin levels and cancer cachexia. High serum adiponectin levels were detected in cachectic patients with colorectal, gastric, pancreas, renal, and prostate cancers (73). However, no differences in adiponectin levels were detected among cachectic and non-cachectic lung cancer patients (73). In a rodent model, plasma adiponectin levels were found to be higher in the early-stage of cachexia and followed with a significant decrease in the late-stage (81). Plasma adiponectin levels were higher in cachectic gastrointestinal cancer patients where adiponectin mRNA expression increased in subcutaneous adipose tissue and remained unaffected in visceral adipose tissue. These findings indicated that subcutaneous adipose tissue is the primary source of plasma adiponectin changes (82). BAT also function as an endocrine organ by secreting factors called “batokines”, including FGF21, IL-6, VEGF-A, and NRG4. Particularly, FGF21 and IL-6 were found to be induced upon BAT activation (83–85). Although the involvement of FGF21 in cancer cachexia needs to be further investigated, a clinical study reported high FGF21 serum levels in elderly patients with cachexia (86). Adipokines are important players in mediating inter-organ crosstalk with adipose tissues and changes in their secretion profile during cancer cachexia would likely influence the metabolism of other tissues. Additional studies are needed to understand roles of adipokines in cachexia progression.

## The Impact of Inflammation and Immune Cells on Adipose Tissue

Several pro-inflammatory cytokines such as TNF-α, IL-6, IL-1, and interferon-γ (IFNγ), either tumor-derived or host-derived, were reported to be upregulated in cachexia (4). Inoculation of tumors overexpressing TNF-α (37), IL-1α (87), IL-6 (88) or IFNγ (89, 90) led to depletion of adipose and muscle tissues. Despite systemic inflammation has been described to be a major player in wasting, clinical therapies targeting a single cytokine so far failed to block cancer cachexia (4). Anti-TNFα therapies failed to prevent weight loss in patients with advanced cancer cachexia (91). While, targeting of IL-6 ameliorates fat loss in cachectic mice (23), clinical trials testing anti-IL-6 therapies are still not satisfactory and warrant further investigation (92–94).

Recently, a direct effect of Toll-Like Receptor-4 (TLR4) disruption on adipose tissue remodeling and cancer cachexia was reported (95). TLR4 signaling was associated with weight loss in LLC tumor-bearing mice where activation of TLR4 signaling induced the release of pro-inflammatory cytokines. Genetic ablation and chemical inhibition of TLR4 signaling reduced the loss of adipose and muscle tissues and prolonged the survival of the tumor-bearing mice. TLR4-deficient mice showed decreased lipolysis and circulating FA. Immune cell composition of WAT was also altered in tumor-bearing TLR4−/− mice where macrophage infiltration in adipose tissue was reduced and macrophage phenotype shifted from M1 to M2. TLR4−/− mice were also resistant to tumor-driven browning of WAT, indicating direct involvement of TLR4 signaling in the metabolic reprogramming in WAT, including lipolysis and browning (95).

Immune cells can secrete cytokines and regulate the microenvironment of tissues. Healthy adipose tissue is characterized by a regulatory type 2 immune signature which suppresses inflammation and maintains adipocyte function (96). On the other hand, gene expression analysis of WAT showed increased levels of activated M2 macrophage markers and unchanged M1 markers, along with a significant increase in the number of macrophages in cachectic mice (19, 21). Adipose tissue immune microenvironment is still not well characterized in cancer cachexia. The role of adaptive immune cells and their influence on metabolic reprogramming during cachexia is unknown while innate immune cells, mainly macrophages, are still being investigated. Interestingly, a protective effect of macrophages in adipose tissue was demonstrated in cachectic mice with hepatocellular carcinoma. These mice with defective myeloid cell activation showed enhanced cachexia-associated adipose tissue loss coincided with decreased number of adipose tissue macrophages, indicating a protective role of immune cells in wasting (97). Conversely, macrophages seemed to contribute to the tissue catabolism observed in murine cachexia models (98). A recent study showed that depletion of local subcutaneous adipose tissue macrophages diminished abundance of UCP-1 and HSL-phosphorylation, subsequently increasing WAT mass in cachectic mice (19). Studies indicate that immune cells can have both anti- and pro-cachectic effects. It is likely that inflammatory cytokines are not sufficient alone to drive cachexia but are part of a complex system regulating the metabolic response leading to cancer cachexia.

## Discussion

Cancer cachexia is a life-limiting disease affecting multiple organs. Recent studies have demonstrated the involvement and impact of adipose tissue in cachexia. Here, we emphasized the multitude of drivers and mechanisms regulating the metabolic reprogramming in adipose tissues during cancer cachexia (**Figure 1**). These metabolic changes lead to a systemic energy imbalance and include inordinate lipolysis and lipogenesis in WAT, activation and expansion of BAT as well as excessive browning of BeAT causing elevated thermogenesis. An interplay between metabolism of adipose tissue and other organs, particularly skeletal muscle, has also been implicated (5). These inter-organ interactions may involve exchange of metabolites and signals such as adipokines and myokines (5). A potential link between adipose tissue lipolysis and skeletal muscle wasting was deciphered using ATGL knockout mice. Lower WAT lipolysis in tumor-bearing ATGL-deficient mice was associated with reduced protein degradation and loss of muscle mass (16). Preserving adipose tissue *via* pharmacological or genetic manipulations affecting lipolysis and thermogenesis protects muscle in murine cachexia models (15, 16, 21, 30, 42, 60). For example, inhibition of GDF15-driven lipid mobilization and oxidation translated into preservation of muscle tissue in cachectic mice (42). Similarly, blocking of PTHrP or the depletion of its receptor in adipocytes in tumor-bearing mice reduced BAT/BeAT thermogenesis and also preserved muscle mass and strength (15, 60). Thus, a crosstalk between metabolism of adipose tissue and skeletal muscle likely exists and this area grants further investigation. One interesting mechanism potentially involved here is the fatty infiltration into skeletal muscle which may contribute to muscle loss. Indeed, a positive correlation between body weight loss and presence of intramyocellular lipid droplets in skeletal muscle of cancer patients was reported (99). Further studies should reveal the role of infiltrating adipocytes in muscle wasting. New therapies against cancer cachexia are urgently needed. Understanding the mechanisms underlying the metabolic dysregulation in adipose tissue and the fat-muscle crosstalk is immensely essential for identification of key players which can be drug targets for future anti-cachexia therapies.

## Author Contributions

All authors listed have made a substantial, direct, and intellectual contribution to the work, read and approved the submitted version for publication.

## Funding

This work was supported by the Scientific and Technological Research Council of Turkey (TUBITAK) grant 120C128 to SK. DA is funded by a TUBITAK-BIDEB scholarship.

## Conflict of Interest

The authors declare that the research was conducted in the absence of any commercial or financial relationships that could be construed as a potential conflict of interest.

## Publisher’s Note

All claims expressed in this article are solely those of the authors and do not necessarily represent those of their affiliated organizations, or those of the publisher, the editors and the reviewers. Any product that may be evaluated in this article, or claim that may be made by its manufacturer, is not guaranteed or endorsed by the publisher.
